# Association of Mutations in the *Melanocortin-2 Receptor Accessory Protein 2* Gene (*MRAP2*) and Obesity: A Systematic Review and Meta-Analysis

**DOI:** 10.3390/ijms27021051

**Published:** 2026-01-21

**Authors:** Ren-Lei Ji, Huifei Sophia Zheng, Alan E. Wilson, Ya-Xiong Tao

**Affiliations:** 1 Department of Anatomy, Physiology and Pharmacology, College of Veterinary Medicine, Auburn University, Auburn, AL 36849, USA; rlj0027@auburn.edu (R.-L.J.); hzz0090@auburn.edu (H.S.Z.); 2 School of Fisheries, Aquaculture, and Aquatic Sciences, Auburn University, Auburn, AL 36849, USA; wilson@auburn.edu

**Keywords:** MRAP2, energy homeostasis, variant, obesity, meta-analysis

## Abstract

Melanocortin-2 receptor accessory protein 2 (MRAP2) is essential for the intricate regulation of energy balance. Although rare *MRAP2* variants have been reported in obese individuals, their overall impact on human obesity risk remains uncertain because previous studies were small, heterogeneous, and often lacked systematic functional characterization. To address this gap, we conducted a comprehensive systematic review and cohort-level meta-analysis to quantify the association between rare coding variants in *MRAP2* and obesity. We systematically searched five major databases (Embase, PubMed, Scopus, Google Scholar, and Web of Science) and identified five eligible publications comprising seven independent cohorts. In total, 27 rare coding *MRAP2* variants were observed in 46 (1.01%) individuals with obesity and 18 (0.34%) individuals with normal weight, among 9771 individuals (5223 with normal weight and 4548 with obesity). Using inverse-variance–weighted random-effects models fitted with restricted maximum likelihood, carriers of rare coding *MRAP2* variants had higher odds of obesity (OR = 2.61; 95% CI, 1.49–4.58; *p* = 8.0 × 10^−4^). Taken together, these findings, derived predominantly from European-ancestry cohorts, support MRAP2 as a biologically plausible susceptibility gene for human obesity and indicate that rare coding *MRAP2* variants are associated with higher odds of obesity, providing a quantitative framework to guide future large-scale genetic and functional studies.

## 1. Introduction

Obesity, an excess of fat accumulation, is a metabolic disorder resulting from the imbalance of food intake and expenditure. Obesity has become a global epidemic, with the World Health Organization reporting that 1.46 billion adults are overweight [1]. In the United States, the National Health and Nutrition Examination Survey indicates that 34.9% of adults are classified as obese and 68.5% are overweight [2]. The increasing prevalence of obesity poses significant public health challenges due to its strong correlation with various chronic diseases, including chronic kidney disease, type 2 diabetes mellitus, metabolic syndrome, nonalcoholic fatty liver disease, cardiovascular disease, and certain cancers [3]. Additionally, the economic burden of obesity is substantial, with national medical costs in the U.S. exceeding $275 billion in 2016 [4]. Despite considerable attention in recent decades, effective therapeutic options for obesity remain limited.

Genetic factors play a crucial role in obesity, with rare variants in over 15 genes identified as causative in monogenic obesity [5,6]. Understanding the genetic underpinnings of obesity is essential for developing new, efficient, and cost-effective methods to address this epidemic. One gene of particular interest is the *melanocortin-2 receptor accessory protein 2* (*MRAP2*), coding a small single transmembrane protein highly expressed in the central nervous system, which modulates energy homeostasis [7,8,9,10]. Targeted deletion of *Mrap2* leads to early-onset severe obesity in mice [11]. MRAP2 interacts with several G protein-coupled receptors (GPCRs), including melanocortin receptors (MCRs) [7], ghrelin receptor (GHSR-1a) [12], orexin receptor 1 (ORX1) [13], prokineticin receptor 1 (PKR1) [14], and several others [15] (reviewed in Ref. [16]). MRAP2 can modulate trafficking, ligand binding and selectivity as well as basal and ligand-stimulated signaling of these receptors [17]. Some of these receptors, especially the melanocortin-4 receptor (MC4R), are involved in modulating food intake and energy balance [18,19,20,21].

Recent studies have identified *MRAP2* variants in both obese and lean individuals, classifying these mutations as pathological, benign, or of unclear effect based on limited clinical and functional evidence [11,22,23,24,25,26,27,28,29,30]. Linking these variants to human obesity has produced inconsistent results, reflecting small sample sizes, heterogeneous ascertainment strategies (severe early-onset versus population-based cohorts), differences in ancestry and obesity definitions, and the modifying effects of incomplete penetrance, gene–gene and gene–environment interactions, sex, age, and genetic heterogeneity.

Meta-analysis, a valuable statistical tool for combining data from individual studies, can address these challenges by enhancing statistical power and the precision of effect size estimates. It has been widely used to assess numerous genes associated with obesity, such as *MC4R* [31], *MC3R* [32], neuropeptide Y [33], leptin and its receptor [34]. However, no large-scale research has focused on *MRAP2* and obesity through meta-analysis to reliably assess gene-disease associations. Therefore, this systematic review and meta-analysis aim to comprehensively evaluate the relationship between genetic variants in the coding region of *MRAP2* and the risk of obesity across multiple population-based studies.

## 2. Materials and Methods

### 2.1. Eligibility Criteria

Any observational studies investigating the association between coding variants in *MRAP2* and obesity were included, with eligibility limited to those published in English (from 2000–2024). Studies were included without restrictions on ethnicity of participants, gender, or age. Using WHO criteria, overweight was defined as body mass index (BMI) at 25–29.9, obesity as BMI ≥ 30, with normal weight defined as BMI 18.5–24.9. For children and adolescents, BMI percentiles were applied: normal weight (5th–85th percentile), overweight (85th–97th percentile), and obesity (≥97th percentile).

The primary meta-analytic outcome was the association between rare coding *MRAP2* variants and obesity (obese vs. normal weight). We pre-specified three additional analytic sets: (i) all individuals with overweight or obesity combined versus normal weight; (ii) children and adolescents only; and (iii) carriers of the recurrent R125C variant versus non-carriers. These secondary and subgroup analyses were treated as hypothesis-generating. Review articles, case reports, in vitro studies only, family-based linkage/segregation studies, and cohorts restricted to severe obesity without an internal control group were excluded. When multiple publications reported data from the same underlying cohort, we included only one publication for descriptive purposes but used cohort-level data as separate observations in the meta-analysis. The protocol for this study was registered beforehand with PROSPERO (Registration Number: CRD42025640633).

### 2.2. Search Strategy

**Figure 1:**
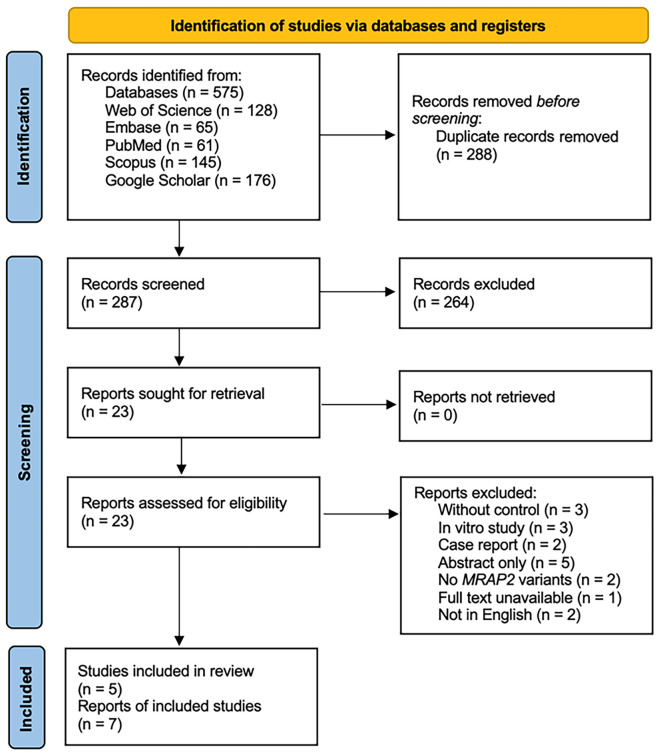
PRISMA flow diagram of study selection. RISMA 2020 flow diagram summarizing identification, screening, eligibility assessment, and inclusion of studies evaluating associations between rare coding *MRAP2* variants and obesity.

A meticulously designed search strategy was developed with the field expert (Tao YX) and a librarian, incorporating a comprehensive set of keywords and controlled vocabulary from pertinent databases. Four databases, including Scopus, PubMed, Embase, and Web of Science, were systematically searched to identify relevant publications. Additionally, Google Scholar was queried to locate any grey literature sources pertaining to the topic (Figure 1). The search was restricted by language and was limited to human studies. Databases were searched from 20 January 2025 to 22 January 2025. Keywords used in the search strategy included combinations of terms, such as melanocortin-2 receptor accessory protein 2 or *MRAP2*, along with variant, mutation, mutant, and loss-of-function, or obesity, overweight, and obese (Supplementary Table S1).

### 2.3. Study Selection

Titles and abstracts were first assessed for eligibility after removing duplicate records using EndNote 20 software (Clarivate, Philadelphia, PA, USA) by two reviewers, individually (Ji RL and Zheng HF). Articles that met the inclusion criteria were chosen for full-text evaluation (Figure 1). Any disagreements during the process were addressed through consensus or by consulting a third reviewer (Tao YX).

### 2.4. Data Extraction

Data were extracted from studies that fulfilled the inclusion criteria using a standardized extraction form (Ji RL and Zheng HF). The following information was recorded from each publication when available: first author, publication year, study design, ethnicity and geographical origin, mean age or age range, mean BMI or BMI range, and ascertainment strategy. For each independent cohort within a publication, we abstracted the numbers of individuals carrying rare coding *MRAP2* variants and non-carriers among obese, overweight, and normal-weight participants. Where reported, age-stratified data (children/adolescents vs. adults) and variant-specific counts (e.g., R125C) were also extracted.

From these 2 × 2 tables, we derived cohort-specific odds ratios (ORs) and corresponding standard errors comparing carriers versus non-carriers for each pre-specified analytic set (all obesity/overweight vs. normal weight; obesity only vs. normal weight; children/adolescents only; and R125C carriers vs. non-carriers). Two authors (Ji RL and Zheng HF) independently performed data extraction and checked all entries; disagreements were resolved through discussion or by consulting a third reviewer (Tao YX).

### 2.5. Meta-Analytic Procedure

Meta-analyses were conducted in R (version 4.0.4, Boston, MA, USA) using the metafor package. The effect measure was the OR for obesity (or obesity/overweight in secondary analyses) comparing carriers versus non-carriers of rare coding *MRAP2* variants within each independent cohort. ORs were log-transformed, and corresponding sampling variances were derived from the extracted 2 × 2 tables using standard formulae. Analyses were performed at the cohort level, so cohorts reported within the same publication contributed separate effect estimates.

We fitted inverse-variance–weighted random-effects models using restricted maximum likelihood (REML) to estimate pooled log ORs. REML was chosen because it provides less biased estimates of the between-study variance (τ^2^) than several alternative estimators, particularly when the number of studies is small and sample sizes are unbalanced, thereby yielding more reliable pooled effects. Study weights were defined as the inverse of the sum of the within-study variance and τ^2^, so that larger and more precise cohorts contributed proportionally more to the pooled estimate.

Pre-specified models were fitted for four analytic sets: (i) obesity only (excluding overweight individuals); (ii) all cohorts including both overweight and obesity; (iii) children and adolescents only; and (iv) carriers of the recurrent R125C variant. Between-study heterogeneity was quantified using τ^2^ and I^2^. To evaluate robustness, we conducted leave-one-out sensitivity analyses for each analytic set by refitting the REML model after omitting each cohort in turn and comparing the resulting pooled log ORs and 95% CIs with the main estimates. Potential small-study effects and publication bias were explored using funnel plots of cohort-level log ORs against their standard errors. Given that only seven cohorts were available, these plots were interpreted descriptively rather than as formal tests of publication bias. All meta-analytic procedures were performed under the guidance of a meta-analysis expert (Wilson AE) to ensure methodological rigor.

## 3. Results

### 3.1. Literature Search

A total of 575 articles were identified from the systematic online search (Figure 1). After removing 288 duplicates, 287 articles remained for title and abstract screening to determine eligibility. Twenty-three articles deemed relevant based on the inclusion criteria were selected for full-text evaluation, and eighteen of those studies were excluded with reasons: Studies without control (*n* = 3), In vitro study (*n* = 3), Case report (*n* = 2), Abstract only (*n* = 5), No *MRAP2* variants (*n* = 2), Full text unavailable (*n* = 1), or Not in English (*n* = 2) (Figure 1). Five independent publications were selected for the meta-analysis [11,23,25,26,35]. The entire process was documented using the PRISMA 2020 Flow Diagram (Figure 1).

### 3.2. Study Characteristics

The details of the five eligible publications were shown in Table 1 and Supplementary Table S2, encompassing a total of 12,236 participants (5223 with normal weight and 7013 with obesity or overweight). Two publications reported data from two independent cohorts each [11,23], yielding seven cohorts in total. These cohorts span different geographic regions, with four focusing on children and adolescents and three on adults and show variation in sex distribution. In all studies, the coding region of *MRAP2* was sequenced to detect variants in both obese and control subjects (Table 1 and Table 2, and Supplementary Table S2).

### 3.3. Relationship Between Coding Variants in MRAP2 and Obesity

*MRAP2* coding variants were identified in 64 (0.91%) subjects with obesity or overweight and 18 (0.34%) individuals with normal weight. Fourteen variants were exclusively found in obese individuals (A3S, E24X, G31V, S15L, N77S, N88Y, E99Q, R113G, S114A, N121S, Q174R, I184T, T193A, and P195L), five were exclusive to normal weight individuals (Q13E, P32L, V91A, H133Y, and D203Y), and four were found in both groups (L115V, R125C, R125H, A137T, and M162T). Additionally, four variants were found in overweight individuals (A3T, F62C, K102X, and P195L) (Supplementary Table S2).

**Figure 2:**
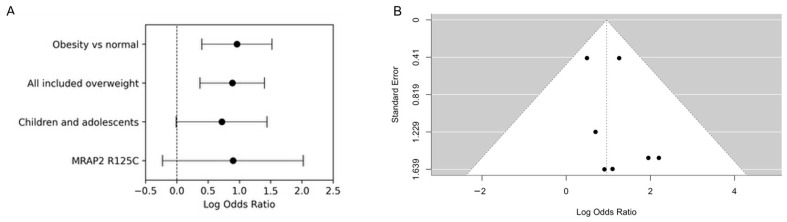
Pooled association between *MRAP2* coding variants and obesity. (**A**) Forest plot of log odds ratios (log ORs) and 95% confidence intervals (CIs) for four pre-specified analytic sets: obesity vs. normal weight, all cohorts including overweight, children/adolescents only, and carriers of the recurrent R125C variant. Effect sizes were estimated using inverse-variance–weighted random-effects models fitted with restricted maximum likelihood (REML); point size is proportional to cohort weight. (**B**) Funnel plot of cohort-level log ORs versus their standard errors for the primary analysis (obesity vs. normal weight) across seven independent cohorts. The vertical dashed line indicates the pooled log OR. With only seven cohorts, the plot is interpreted descriptively and is not used as a formal test of publication bias.

**Figure 3:**
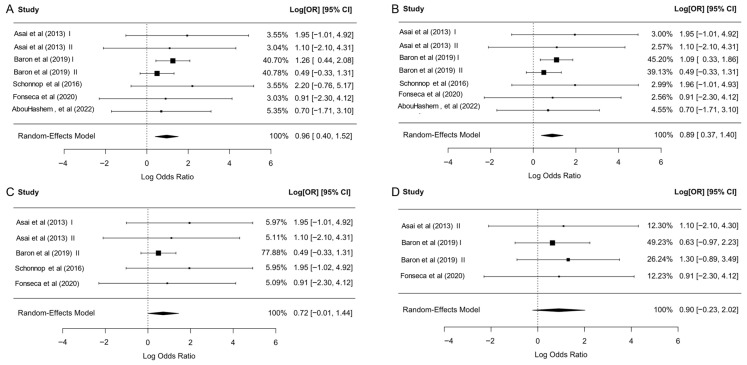
Study-specific associations between *MRAP2* variants and obesity in different analytic sets. Forest plots showing cohort-level log ORs and 95% CIs, together with REML random-effects pooled estimates, for: (**A**) all studies after excluding overweight individuals, (**B**) all cohorts including overweight, (**C**) children and adolescents only, and (**D**) carriers of the R125C variant. Squares represent cohort log ORs (area proportional to inverse-variance weight); horizontal lines represent 95% CIs; diamonds represent pooled estimates. Asai et al. (2013) I and II [11]. Baron et al. (2019) I and II [23]. Schonnop et al. (2016) [35]. Fonseca et al. (2020) [25]. AbouHashem et al. (2022) [26].

Pooling all reported rare coding *MRAP2* variants in 7013 obese or overweight subjects and 5223 normal-weight subjects revealed a significant positive association between these variants and adiposity (OR = 2.43; 95% CI, 1.45–4.06; *p* = 7.0 × 10^−4^; I^2^ = 0%; τ^2^ = 0) (Table 3, Figure 2A and Figure 3). When the analysis was restricted to individuals with obesity versus normal weight, the association was slightly stronger (OR = 2.61; 95% CI, 1.49–4.58; *p* = 8.0 × 10^−4^), with low heterogeneity (I^2^ = 3.68%; τ^2^ = 0.03) (Table 3, Figure 2A and Figure 3).

Given the tendency of monogenic obesity to present early [39], typically during childhood, we narrowed our scope to include only children and adolescents. One study included adults who developed obesity in childhood [25]; therefore it was included in the children and adolescent group. In this subgroup, *MRAP2* variants were identified in 24 (1.04%) obese and 9 (0.41%) normal weight subjects, yielding a pooled OR of 2.05 (95% CI, 0.99–4.24; *p* = 0.05; I^2^ = 0%; τ^2^ = 0) in 2310 obese and 2190 normal-weight children and adolescents. This borderline association, with a confidence interval that includes the null, should be interpreted cautiously (Table 3, Figure 2A and Figure 3).

Since the MRAP2 R125C variant was reported in four studies, we further analyzed the relationship between this mutation and obesity (Supplementary Table S2). R125C was identified in 12 (0.19%) obese and 3 (0.07%) normal weight subjects. The pooled OR was 2.45 (95% CI, 0.80–7.45; *p* = 0.12; I^2^ = 0%; τ^2^ = 0), indicating a positive but statistically non-significant association with wide CIs that reflect limited precision (Table 3, Figure 2A and Figure 3) and suggesting that the current data are compatible with both a moderate increase in risk and no effect.

The funnel plot of cohort-level log ORs (Figure 2B and Supplementary Figure S1) showed no obvious outliers or strong asymmetry; however, with only seven cohorts, this plot is descriptive and underpowered for a formal assessment of publication bias.

Leave-one-out sensitivity analyses supported the robustness of the primary findings (Supplementary Tables S3–S6). For the “all cohorts including overweight” model, omitting each cohort in turn yielded pooled log ORs between 0.72 and 1.14, with all 95% CIs remaining above 0 and *p*-values ≤ 0.042. For the “obesity vs. normal weight” model, leave-one-out log ORs ranged from 0.73 to 1.28, again with all 95% CIs excluding the null and *p*-values ≤ 0.039. Similar ranges were obtained when each of the two Baron et al. cohorts [23] was excluded individually, indicating that the overall association is not driven by a single study despite the substantial contribution of these cohorts to the total number of mutation carriers. In contrast, leave-one-out analyses for the children/adolescents-only and R125C models yielded consistently positive but non-significant pooled log ORs with wide CIs crossing the null, underscoring the exploratory and imprecise nature of these subgroup estimates.

**Figure 4:**
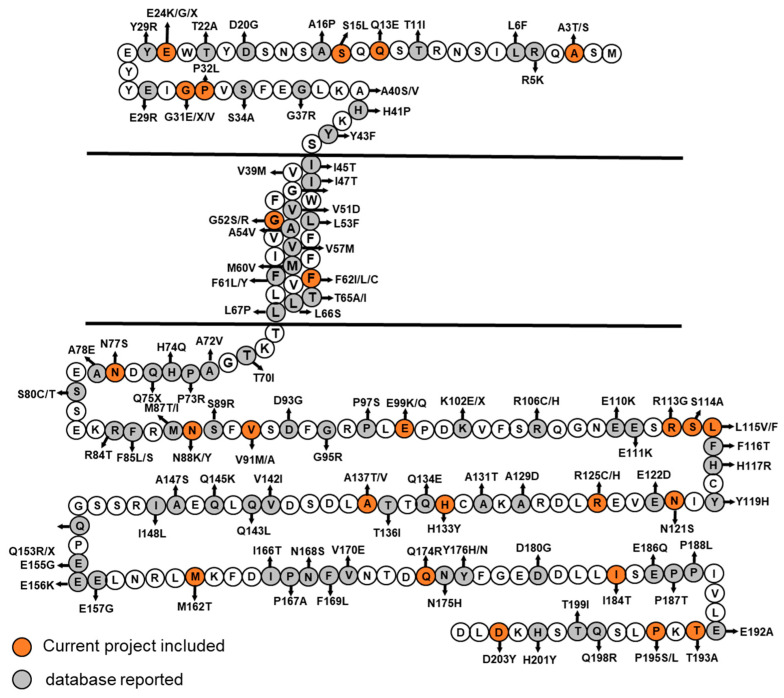
Naturally occurring *MRAP2* coding variants. Schematic representation of the MRAP2 protein showing the positions of rare coding variants. Orange circles denoted variants identified in the current meta-analysis; grey circles denoted additional variants reported in the gnomAD v4.1.0 database (accessed on 26 January 2025). Variants with functional data or predicted pathogenicity were listed in Table 2.

Various *MRAP2* variants are documented in the gnomAD v4.1.0 database (https://gnomad.broadinstitute.org/, accessed on 26 January 2025) [40]. Most variants identified in this study were found in gnomAD v4.1.0 with allele frequencies ≤ 0.1% (Table 2). We illustrated the combined *MRAP2* mutations identified in this study along with those reported in databases in Figure 4.

We further classified these variants based on their functional characteristics and/or analysis using AlphaMissense (https://alphafold.ebi.ac.uk/entry/Q96G30, accessed on 26 January 2025) [38]. Where available, published in vitro data on the impact of individual MRAP2 variants on MC4R trafficking and signaling were also taken into account (Table 2). Eleven variants were categorized as likely pathogenic, including E24X, P32L, F62C, N77S, N88Y, K102X, R113G, N121S, R125C, Q174R and P195L (Table 2). Four variants with conflicting evidence (A3T, G31V, T193A, and L115V) were classified as variants of uncertain significance. The remaining variants were categorized as likely benign or benign (Table 2). Variants with marked or partial impairment of MC4R signaling tended to be reported in obese probands, whereas variants with preserved MC4R function were observed in both obese and normal-weight subjects (Table 2).

## 4. Discussion

Our meta-analysis indicates that rare coding variants in *MRAP2* were associated with higher odds of obesity, consistent with findings in a mouse model where *Mrap2^−/−^* mice exhibit early-onset severe obesity [11]. This is the first meta-analysis to investigate the association between *MRAP2* variants and obesity across various ethnic populations. This underscores the need for large-scale research to reliably assess gene-disease associations.

Obesity is a multifactorial disorder, but more than 15 gene mutations can result in monogenic obesity in humans, such as *MC4R* [5,18,20,41]. Our study highlights the significance of examining relevant candidate genes in large-scale case–control studies to uncover rare variants with monogenic effects [31,32,42]. Identifying these mutations is crucial for explaining the missing heritability of obesity and developing personalized prevention and treatment strategies for high-risk individuals [43,44].

Our study found a significant association in the overall obesity vs. normal weight analysis (OR = 2.61; 95% CI, 1.49–4.58; *p* = 8.0 × 10^−4^) and a borderline, suggestive association in children or adolescents alone (OR = 2.05; 95% CI, 0.99–4.24; *p* = 0.05), with the latter result requiring cautious interpretation given that the 95% CI includes 1.0. This aligns with previous study showing that rare *MRAP2* variants are associated with higher BMI and obesity risk in both adults and children [23]. Utilizing exome sequencing data from large database (the Accelerating Medicine Partnership Type 2 Diabetes Knowledge Portal), it was shown that missense or protein-truncating *MRAP2* mutations (with MAF < 1%) are significantly associated with higher BMI (*p* = 3.49 × 10^−4^ with b = 0.0364 kg m^−2^, 95% CI: 0.0165–0.0564 kg m^−2^) and have a stronger association (*p* = 2.36 × 10^−5^ with b = 0.154 kg m^−2^, 95% CI: 0.0828–0.226 kg m^−2^) in deleterious missense or protein-truncating *MRAP2* mutations [23].

Large-scale research has also found positive associations between obesity and other gene mutations, such as *MC3R* and *MC4R*. A meta-analysis of *MC3R* showed a strong link between rare coding variants that cause partial/complete loss-of-function and obesity (OR = 3.07; 95% CI, 1.48–7.00; *p* = 4.2 × 10^−3^) [32]. The *MC4R* variant rs17782313 is significantly associated with obesity under both the allele contrast model and the dominant model [45]. Furthermore, meta-analysis confirm that loss-of-function variants in *MC4R* do not show a connection with binge eating disorder [46,47], different from conclusion in a controversial study [48]; however, gain-of-function variants in *MC4R* are linked to binge eating disorder [31,49].

In vitro functional assays on MRAP2 variants support its involvement in obesity pathogenesis [11,23,28,37]. MRAP2 variants disrupt MC4R and MC3R signaling. Variants such as G31V, G52R, F62C, N77S, N88Y, R125C, K102X, and P195L in MRAP2 have been shown to reduce ligand (α-MSH and/or ACTH)-induced cAMP signaling on MC4R [23,28]. Several MRAP2 mutants, including G31V, F62C, N88Y, R113G, S114A, L115V, N121S, R125C, T193A, have been shown to exhibit distinct defects in [Nle^4^,D-Phe^7^]-MSH-induced cAMP and/or inositol phosphate signaling pathways [36]. Two mutants in MRAP2 (N88Y and R125C) impair α-MSH-induced cAMP signaling on MC3R [37]. Moreover, MRAP2 mutants may affect pathways outside of MCR signaling, such as ghrelin receptor, orexin receptor, and PKR1 signaling [7,12,13,14]. Loss-of-function MRAP2 variants are also implicated in hypertension and hyperglycemia [23]. Taken together, these data reinforce the biological plausibility that disruption of MRAP2-mediated GPCR signaling contributes to obesity and related metabolic disturbances, but the current functional evidence is incomplete and should be regarded as hypothesis-generating.

The R125 residue in MRAP2 has been identified as crucial for its function [50]. Two common MRAP2 variants, R125C and R125H, have been identified in both obese and normal weight individuals (Supplementary Table S2). R125C has been shown to impairs signaling of MC3R, MC4R, and PKR1 [37,50], indicating the R125C variant as “likely pathogenic”. Our results also indicated a positive association between the *MRAP2* R125C and obesity, with an OR of 2.45 and a non-significant *p*-value of 0.12 and a wide 95% CI (0.80–7.45) (Table 3), suggesting that this estimate is compatible with both a moderate effect and no effect. Different genetic backgrounds and lifestyle modifications may contribute to the varying phenotypes observed in individuals harboring *MRAP2* variants. Similar phenomena have been reported in *MC4R* variants [47,48,51,52]. These findings highlight the need for further research.

The strengths of this study lie in the originality and overall statistical power of our meta-analytic approach. Potential bias was mitigated by independent dual screening and data extraction. Nevertheless, several limitations should be acknowledged. First, most participants are of European ancestry and allele frequencies differ across populations; consequently, the pooled estimates largely reflect European cohorts and may not generalize to under-represented groups, limiting ancestry-specific inference. Second, functional annotation is incomplete for many *MRAP2* variants, which constrains our ability to perform stratified meta-analyses by functional impact and to draw firm mechanistic conclusions. Third, although between-study heterogeneity was low (I^2^ ≤ 3.7% across all models), many odds ratio estimates have wide 95% CIs, especially for rare variants, indicating limited precision and the possibility that some effect sizes are inflated. The pooled result is also heavily influenced by the largest European cohort [23], so the robustness of the association remains partly dependent on a single study. However, leave-one-out sensitivity analyses showed that the primary pooled association for obesity versus normal weight remained of similar magnitude and statistically significant when each cohort, including the two Baron et al. cohorts [23], was omitted in turn, suggesting that our main findings are not solely driven by a single cohort, although they should still be interpreted in the context of predominantly European-ancestry data. The significant pooled association in our primary analysis therefore reflects the aggregated, gene-level effect of multiple rare coding variants, whereas the number of carriers for any individual variant is small and variant-specific odds ratios remain imprecise and hypothesis-generating.

Finally, we were unable to incorporate disease-related mechanistic and clinical covariates—such as comorbidities, lifestyle factors, glycemic and blood-pressure traits, or detailed endocrine and receptor-signaling measures—because these variables were not consistently reported across studies. Residual confounding by these unmeasured factors is therefore likely and may contribute to the observed associations. Future validation in large, ancestrally diverse cohorts with harmonized phenotyping, such as the UK Biobank and other population-based resources, will be essential to refine effect-size estimates, assess reproducibility, and link MRAP2 variants more directly to pathophysiological pathways.

## 5. Conclusions

In summary, this systematic review and meta-analysis indicates that rare coding variants in *MRAP2* are associated with higher odds of obesity in the currently available observational studies. Because these variants are rare and effect estimates remain imprecise, our findings should be viewed as hypothesis-generating rather than definitive evidence of causality or immediate clinical utility. Nonetheless, the results support *MRAP2* as a biologically plausible candidate gene for monogenic and oligogenic forms of obesity and provide a quantitative framework for future work. Large, ancestrally diverse cohorts that integrate detailed mechanistic phenotyping and functional characterization of MRAP2 variants will be required to clarify causality, delineate underlying pathways, and evaluate any potential role in genetic risk stratification or targeted interventions.

## Figures and Tables

**Table 1:** Characteristics of studies included in the meta-analysis.

First Author (Year)		Geographic Origin	Sex Ratio (M/F)	Age	BMI or Percentage	Carriers/Total
Asai et al. [11]						
Cohort I	Normal	Swedish	NR	15–20	20–25	0/376
	Obese		NR	6–21	>30 (3)	1/376
Cohort 2	Normal	UK	NR	<16	5–85th	0/488
	Obese		NR	<16	≥97th	3/488
Baron et al. [23]						
Cohort I	Normal	Europe	1177/1606	47.2 (11.7)	22.3 (1.83)	8/2783
	Obese		618/1373	49.0 (12.7)	40.2 (8.76)	20/1991
	Overweight		1577/908	52.4 (12.0)	27.2 (1.39)	18/2465
Cohort 2	Normal		540/502	17.5 (3.30)	20.2 (2.32)	9/1042
	Obese		539/598	12.3 (2.35)	31.0 (5.56)	16/1137
Schonnop et al. [35]						
	Normal	Germany	88/96	children and adolescents	5–85th	0/184
	Obese		86/98		≥97th	3/184
da Fonseca et al. [25]						
	Normal	Brazil	NR	NR	18.5 ≤ BMI ≤ 24.9	0/100
	Obesity		25/97	37.0 (28; 45)	47.0 (42.8; 52.6)	1/122
AbouHashem et al. [26]	Normal	Qatar	160/340	40.5 (12.1)	22.6 ± 1.7	1/250
	Obesity				40.3 ± 3.7	2/250

**Table 2:** Pathogenicity prediction for the human *MRAP2* variants.

Variants	Reference Identifying Variants	Function Characterization for MC4R Signaling	Allele Frequency (gnomAD)	Pathogenicity (Based on Functional Assay)	Pathogenicity (Based on AlphaMissense)
A3T	[23]	Reduced [23]	1.3 × 10^−5^	Uncertain Significance	Likely benign
A3S	[23]	WT-like [23]	6.2 × 10^−7^	Benign	Likely benign
Q13E	[23]	Increased [23]	6.2 × 10^−7^	Benign	Likely benign
S15L	[26]	NA	2.8 × 10^−5^	NA	Likely benign
E24X	[11]	NA	4.9 × 10^−6^	NA	Likely Pathogenic
G31V	[23]	Reduced [23]; Increased [36]	NA	Uncertain Significance	Uncertain
P32L	[23]	Reduced [23]; WT-like [36]	1.8 × 10^−6^	Uncertain Significance	Likely Pathogenic
F62C	[23]	Reduced [23,36]	NA	Likely Pathogenic	Likely Pathogenic
N77S	[23]	Reduced [23]	2.1 × 10^−5^	Likely Pathogenic	Likely benign
N88Y	[11]	Reduced [36,37]	1.8 × 10^−5^	Likely Pathogenic	Likely benign
V91A	[23]	Increased [23]; WT-like [36]	3.1 × 10^−6^	Benign	Likely benign
E99Q	[23]	WT-like [23]	6.2 × 10^−6^	Benign	Likely benign
K102X	[23]	Reduced [23]	6.1 × 10^−7^	Likely Pathogenic	Likely Pathogenic
R113G	[23]	Reduced [23,36]	9.2 × 10^−6^	Likely Pathogenic	Likely benign
S114A	[23]	Increased [23]	NA	Benign	Likely benign
L115V	[11,26]	Reduced [36]; WT-like [37]	4.3 × 10^−5^	Uncertain Significance	Likely benign
N121S	[23]	Reduced [23,36]	6.2 × 10^−7^	Likely Pathogenic	Likely benign
R125C	[11,23,25]	Reduced [23,36,37]	4.2 × 10^−4^	Likely Pathogenic	Likely benign
R125H	[23,35]	Increased [23] or WT-like [35]	1.0 × 10^−3^	Benign	Likely benign
H133Y	[23]	Increased [23] or WT-like [36]	1.8 × 10^−6^	Benign	Likely benign
A137T	[23,35]	WT-like [35]; Increased [23]	7.4 × 10^−6^	Benign	Likely benign
M162T	[23]	Increased [23]	1.7 × 10^−5^	Benign	Likely benign
Q174R	[35]	Reduced [35]	6.3 × 10^−5^	Likely Pathogenic	Likely benign
I184T	[26]	NA	NA	NA	Likely Pathogenic
T193A	[23]	Increased [23]; Reduced [36]	1.2 × 10^−6^	Uncertain Significance	Likely benign
P195L	[23]	Reduced [23]	2.4 × 10^−6^	Likely Pathogenic	Likely benign
D203Y	[23]	Increased [23]	1.4 × 10^−5^	Benign	Likely benign

Pathogenicity classification based on functional assays categorizes variants as follows: Likely Pathogenic: Meets strong evidence criteria with additional supporting data. Uncertain Significance: Lacks sufficient evidence or presents conflicting data. Benign: Requires strong and consistent evidence to confirm benignity. Pathogenicity classification based on AlphaMissense categorizes variants as ‘likely pathogenic,’ ‘likely benign,’ or ‘uncertain’ based on amino acid sequence predictions. This approach leverages models developed from AlphaFold2 (https://alphafold.ebi.ac.uk/entry/Q96G30) [38] (accessed on 25 January 2025). WT, wild type; NA, not available.

**Table 3:** Odds ratios of obesity among carriers of *MRAP2* coding variants, pooling results based on all eligible studies.

Obesity vs. Normal	Obesity	Normal Weight	OR (95% CI)	*p* Value	*I* ^2^	τ^2^
Carriers of coding variants	46 (1.01%)	18 (0.34%)	2.61 (1.49–4.58)	8.0 × 10^−4^	3.68%	0.03
Non-carriers of coding variants	4502 (98.99%)	5205 (99.66%)				
All included overweight						
Carriers of coding variants	64 (0.91%)	18 (0.34%)	2.43 (1.45–4.06)	7.0 × 10^−4^	0.00%	0
Non-carriers of coding variants	6949 (99.09%)	5205 (99.66%)				
Children						
Carriers of coding variants	24 (1.04%)	9 (0.41%)	2.05 (0.99–4.24)	0.05	0.00%	0
Non-carriers of coding variants	2286 (98.96%)	2181 (99.59%)				
R125C						
Carriers of coding variants	12 (0.19%)	3 (0.07%)	2.45 (0.80–7.45)	0.12	0.00%	0
Non-carriers of coding variants	6191 (99.81%)	4410 (99.93%)				

## Data Availability

The raw data supporting the conclusions of this article will be made available by the authors upon request, without undue reservation.

## Supplementary Materials

The following supporting information can be downloaded at https://www.mdpi.com/article/10.3390/ijms27021051/s1.

## Abbreviations

The following abbreviations are used in this manuscript:

MRAP2	Melanocortin-2 receptor accessory protein 2
GPCR	G protein-coupled receptor
MCR	Melanocortin receptor
MC4R	melanocortin-4 receptor
MC3R	melanocortin-3 receptor
GHSR-1a	Ghrelin receptor
ORX1	Orexin receptor 1
PKR1	Prokineticin receptor 1
BMI	Body mass index
OR	Odds ratio
CI	Confidence interval
REML	Restricted Maximum Likelihood
WT	Wild type
ACTH	Adrenocorticotropic hormone
α-MSH	α-Melanocyte-stimulating hormone
