# Supplementary material for: Association of Mutations in the Melanocortin-2 Receptor Accessory Protein 2 Gene (MRAP2) and Obesity: A Systematic Review and Meta-Analysis
Source: Int J Mol Sci. 2026 Jan 21;27(2):1051. doi: 10.3390/ijms27021051 (PMC12841619; doi:10.3390/ijms27021051)
Supplement: Supplementary file 1 [file ijms-27-01051-s001.zip › ijms-4049188-supplementary.pdf]

Association of mutations in the *melanocortin-2 receptor accessory protein 2* gene (*MRAP2*) and obesity: a systematic review and meta-analysis

Ren-Lei Ji[1], Huifei Sophia Zheng[1], Alan E. Wilson[2], Ya-Xiong Tao[1*]

[1] Department of Anatomy, Physiology and Pharmacology, College of Veterinary Medicine, Auburn University, Auburn, AL, USA

[2] School of Fisheries, Aquaculture, and Aquatic Sciences, Auburn University, Auburn, AL, USA

*Corresponding author (email: taoyaxi@auburn.edu)

Supplementary Table 1. Search Strategies

20 January 2025

Database: Embase

‘Melanocortin 2 receptor accessory protein 2’ AND ‘mutations’

'mrap2 gene'

'mrap2 gene' AND 'variant'

'mrap2 gene' AND 'obesity'

'mrap2 gene' AND 'mutation'

‘Melanocortin 2 receptor accessory protein 2’

Database: Scopus

'mrap2' AND ' mutations'

'mrap2' AND ' variants '

'mrap2' AND ' obesity '

'mrap2' AND ' obese '

'mrap2'

Database: Web of Science

‘Melanocortin 2 receptor accessory protein 2’ AND ‘obesity’

‘Melanocortin 2 receptor accessory protein 2’ AND ‘variant’

‘Melanocortin 2 receptor accessory protein 2’ AND ‘variant’

'mrap2' AND ' mutations'

'mrap2' AND ' variants '

'mrap2' AND ' obesity '

'mrap2' AND ' obese '

Database: PubMed

'mrap2' &' variant '

'mrap2' & ' mutations'

'mrap2' & ' obesity '

‘Melanocortin 2 receptor accessory protein 2’ & ‘obesity’

‘Melanocortin 2 receptor accessory protein 2’ & ‘variant’

‘Melanocortin 2 receptor accessory protein 2’ & ‘obese’

Database: Google Scholar

Melanocortin 2 receptor accessory protein 2’ & ‘variant’

Melanocortin 2 receptor accessory protein 2’ & ‘mutation’

Melanocortin 2 receptor accessory protein 2’ & ‘obesity’

'MRAP2' & 'variant'

'MRAP2' & 'obesity'

'MRAP2' & 'mutation'

Supplementary Table 2 Mutations detected in included studies

	Mutation	No of carriers with mutations in obesity	No of carriers	No of carriers with mutations in normal weight	No of Carriers in normal weight	Sex/age/BMI/percentile for age
Asai *et al.* (1)						
Cohort I	E24X	1	488	0	488	M/19/63
	L115V	1	488	0	488	M/5/24
	R125C	1	488	0	488	F/8/29
Cohort II	N88Y	1	376	0	376	M/11/29.6
Schonnop *et al.* (2)	R125H	1	184	0	184	F/12.5/25.6/98th
	A137T	1	184	0	184	M/15.7/29.8/ 99th
	Q174R	1	184	0	184	F/7.9/23.92/97th
Baron *et al.* (3)						
Cohort I	A3T	1	4456	0	2783	
	A3S	1	4456	0	2783	
	G31V	1	4456	0	2783	F/48/30.1
	F62C	1	4456	0	2783	M/44/25.3
	N77S	1	4456	0	2783	M/43/49.6
	E99Q	1	4456	0	2783	
	K102*	1	4456	0	2783	F/61/25.3
	R113G	1	4456	0	2783	
	N121S	1	4456	0	2783	
	R125C	8	4456	2	2783	
	R125H	19	4456	3	2783	
	A137T	0	4456	1	2783	
	M162T	1	4456	1	2783	
	T193A	1	4456	0	2783	
	P195L	2	4456	0	2783	F/61/32.8;F49/27.9
	D203Y	0	4456	1	2783	
Cohort II	A3S	1	1137	0	1042	
	Q13E	0	1137	1	1042	
	P32L	0	1137	1	1042	
	N77S	1	1137	0	1042	M/12/30.9/99th
	V91A	0	1137	1	1042	
	R113G	1	1137	0	1042	
	S114A	1	1137	0	1042	
	R125C	4	1137	1	1042	
	R125H	6	1137	3	1042	
	H133Y	0	1137	1	1042	
	A137T	0	1137	1	1042	
	P195L	0	1137	1	1042	F/17/29.7/95th
da Fonseca *et al.* (4)	R125C	1	122	0	100	M/37.8/41.8

AbouHashem *et al*. (5)	S15L	1	250	0	250	M/39/40.6
	I184T	1	250	0	250	M/35/40.8
	L115V	0	250	1	250	M/39/23.9

Supplementary Table S3. Leave-one-out sensitivity analysis for the association between rare MRAP2 coding variants and obesity (obesity vs. normal weight).

study_removed	estimate	pval	ci.lb	ci.ub	tau^2	I^2
Asai et al (2013) I	0.9233	0.0017	0.347	1.4996	0.0296	4.6576
Asai et al (2013) II	0.958	0.0013	0.3747	1.5413	0.0367	5.7791
Baron et al (2019) I	0.7316	0.0387	0.0379	1.4254	0	0
Baron et al (2019) II	1.2777	0.0003	0.5834	1.972	0	0
Schonnop et al (2016)	0.9135	0.0018	0.3412	1.4859	0.0263	4.1605
Fonseca et al (2020)	0.9646	0.0012	0.38	1.5492	0.0379	5.9518
AbouHashem et al (2022)	0.9832	0.0015	0.3764	1.5899	0.0504	7.4336

Values are from REML random-effects models fitted to cohort-level log odds ratios (log ORs). Each row shows the pooled effect obtained after omitting the indicated cohort from the meta-analysis. estimate denotes the pooled log OR; p-value for the null hypothesis of no association; ci.lb and ci.ub, the lower and upper bounds of the 95% confidence interval; tau^2, the estimated between-study variance; I^2, the percentage of total variability attributable to between-study heterogeneity.

Supplementary Table S4. Leave-one-out sensitivity analysis for the association between rare MRAP2 coding variants and adiposity when overweight and obesity are combined (all cohorts including overweight).

study_removed	estimate	pval	ci.lb	ci.ub	tau^2	I^2
Asai et al (2013) I	0.8548	0.0013	0.3334	1.3763	0	0
Asai et al (2013) II	0.8821	0.0009	0.3618	1.4024	0	0
Baron et al (2019) I	0.7183	0.0424	0.0246	1.4121	0	0
Baron et al (2019) II	1.1411	0.0007	0.4828	1.7994	0	0
Schonnop et al (2016)	0.8546	0.0013	0.3332	1.3761	0	0
Fonseca et al (2020)	0.8872	0.0008	0.3669	1.4075	0	0
AbouHashem et al (2022)	0.8968	0.0008	0.3711	1.4225	0	0

Description as in Supplementary Table S3. This model includes both overweight and obese individuals versus normal weight as the outcome definition.

Supplementary Table S5. Leave-one-out sensitivity analysis for the association between rare MRAP2 coding variants and obesity in children and adolescents.

study_removed	estimate	pval	ci.lb	ci.ub	tau^2	I^2
Asai et al (2013) I	0.641	0.0927	-0.106	1.3882	0	0
Asai et al (2013) II	0.6987	0.0656	-0.045	1.4425	0	0
Baron et al (2019) II	1.5137	0.0541	-0.027	3.0541	0	0
Schonnop et al (2016)	0.6417	0.0923	-0.105	1.3888	0	0
Fonseca et al (2020)	0.7091	0.0617	-0.035	1.4528	0	0

Column definitions are as in Supplementary Table S3. This analysis is restricted to cohorts of children and adolescents, using age-appropriate BMI percentile cut-offs for normal weight and obesity.

Supplementary Table S6. Leave-one-out sensitivity analysis for the association between the recurrent R125C variant in MRAP2 and obesity.

study_removed	estimate	pval	ci.lb	ci.ub	tau^2	I^2
Asai et al (2013) II	0.869	0.1556	-0.331	2.0685	0	0
Baron et al (2019) I	1.1583	0.1498	-0.418	2.7348	0	0
Baron et al (2019) II	0.7537	0.2587	-0.554	2.0617	0	0
Fonseca et al (2020)	0.8959	0.1431	-0.303	2.095	0	0

Column definitions are as in Supplementary Table S3. This analysis includes only cohorts that reported data for the recurrent R125C variant, comparing carriers versus non-carriers.

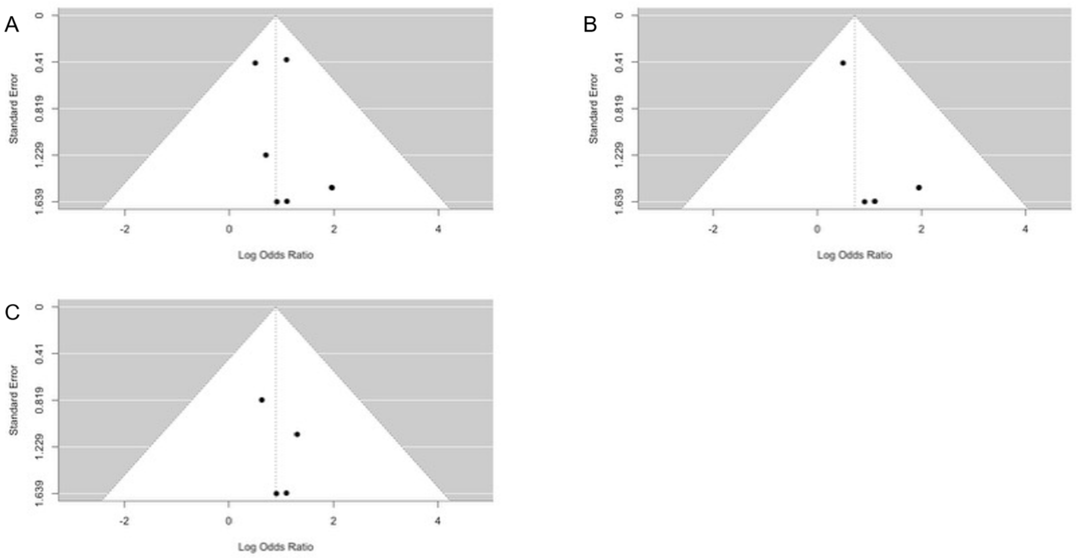

Supplementary Figure 1. Funnel plots of the association between *MRAP2* mutations and obesity. Funnel plots of cohort-level log ORs versus their standard errors for the four analytic sets: (A) all cohorts including overweight, (B) children/adolescents only, and (C) carriers of the R125C variant. Vertical dashed lines indicate the corresponding REML random-effects pooled log ORs. Given the small number of cohorts, these plots are presented for descriptive purposes only and are not used as formal tests of publication bias.

References

1. Asai M, Ramachandrappa S, Joachim M, Shen Y, Zhang R, Nuthalapati N, Ramanathan V, Strochlic DE, Ferket P, Linhart K, Ho C, Novoselova TV, Garg S, Ridderstrale M, Marcus C, Hirschhorn JN, Keogh JM, O'Rahilly S, Chan LF, Clark AJ, Farooqi IS, Majzoub JA. Loss of function of the melanocortin 2 receptor accessory protein 2 is associated with mammalian obesity. Science. 2013;341(6143):275-278.
2. Schonnop L, Kleinau G, Herrfurth N, Volckmar AL, Cetindag C, Müller A, Peters T, Herpertz S, Antel J, Hebebrand J, Biebermann H, Hinney A. Decreased melanocortin-4 receptor function conferred by an infrequent variant at the human melanocortin receptor accessory protein 2 gene. Obesity. 2016;24(9):1976-1982.
3. Baron M, Maillet J, Huyvaert M, Dechaume A, Boutry R, Loiselle H, Durand E, Toussaint B, Vaillant E, Philippe J, Thomas J, Ghulam A, Franc S, Charpentier G, Borys JM, Levy-Marchal C, Tauber M, Scharfmann R, Weill J, Aubert C, Kerr-Conte J, Pattou F, Roussel R, Balkau B, Marre M, Boissel M, Derhourhi M, Gaget S, Canouil M, Froguel P, Bonnefond A. Loss-of-function mutations in MRAP2 are pathogenic in hyperphagic obesity with hyperglycemia and hypertension. Nat Med. 2019;25(11):1733-1738.
4. da Fonseca ACP, Abreu GM, Zembrzuski VM, Campos Junior M, Carneiro JRI, Nogueira Neto JF, Magno F, Rosado EL, Bozza PT, de Cabello GMK, Cabello PH. Study of LEP, MRAP2 and POMC genes as potential causes of severe obesity in Brazilian patients. Eat Weight Disord. 2021;26(5):1399-1408.
5. AbouHashem N, Zaied RE, Al-Shafai K, Nofal M, Syed N, Al-Shafai M. The spectrum of genetic variants associated with the development of monogenic obesity in Qatar. Obes Facts. 2022;15(3):357-365.
